# Malignant Lymphoma in the Parasellar Region

**DOI:** 10.1155/2014/747280

**Published:** 2014-02-10

**Authors:** Takao Koiso, Hiroyoshi Akutsu, Shingo Takano, Tetsuya Yamamoto, Eiichi Ishikawa, Yasushi Okoshi, Akira Matsumura

**Affiliations:** ^1^Department of Neurosurgery, Faculty of Medicine, University of Tsukuba, Tennodai 1-1-1, Tsukuba, Ibaraki 305-8575, Japan; ^2^Department of Hematology, Faculty of Medicine, University of Tsukuba, Tennodai 1-1-1, Tsukuba, Ibaraki 305-8575, Japan

## Abstract

The entity of pituitary (sellar or parasellar) lymphoma includes primary pituitary lymphoma (PPL) and secondary pituitary lymphoma (SPL). The latter has an involvement of systemic lymphoma. Both of these lymphomas are extremely rare. We describe a patient with SPL showing a good prognosis. A 78-year-old woman presented with diplopia, left ptosis, and back pain. Magnetic resonance (MR) imaging revealed a parasellar mass lesion extending to the upper clivus and another mass lesion with compression fracture of the Th3 vertebral body. Transsphenoidal exploration was performed, and it showed diffuse large B-cell lymphoma. Based on the positive tumor cells in the following bone marrow aspiration and hepatosplenomegaly in computed tomography (CT) findings, this patient was diagnosed as having a pituitary involvement of systemic lymphoma. After chemotherapy, she achieved complete remission for 4 years. The entity of pituitary lymphoma is extremely rare. Nineteen cases of PPL and 16 cases of SPL have been reported. Generally, clinical and radiological diagnosis was difficult because there are no specific findings. Therefore, biopsy was necessary in all of the cases. T2 hypointensity of a lesion in MR imaging in addition to an elevated serum level of soluble interleukin-2 receptor (sIL-2R) in a patient with a sellar lesion can be useful clues for the differential diagnosis of this rare disease.

## 1. Introduction

According to previous reports, the entity of pituitary (sellar or parasellar) lymphoma includes primary pituitary lymphoma (PPL) and secondary pituitary lymphoma (SPL). SPL includes the involvement of systemic lymphoma. Both of these lymphomas are extremely rare. Nineteen cases of PPL [1–17] and 16 cases of SPL [13, 18–30] have been reported. We describe a patient with SPL treated with chemotherapy following transnasal biopsy and showing a good prognosis.

## 2. Case Description

A 78-year-old woman with no remarkable medical history presented with diplopia, left ptosis, and back pain that has persisted for over one month. Her ocular motility improved spontaneously, but 3 months later, fever, polyuria, and polydipsia appeared. Her body temperature rose to between 38° and 39°C every night. Her urinary volume was 4-5 L/day. There was no weight loss or neurological abnormality. So she went to general hospital and performed brain and thoracic magnetic resonance (MR) imaging. These images showed double lesion at sellar region and Th3 vertebral body. Then, she was admitted to our hospital.

Laboratory studies showed anemia and a slight elevation of the serum levels of inflammatory markers. Hemoglobin was 10.3 g/dL (normal, 12.0–16.0). C-reactive protein (CRP) was 4.09 g/mL (normal, <5 mg/dL), and the erythrocyte sedimentation rate (ESR) was 20 mm/hr (normal, <3–15 mm/hr). The lactate dehydrogenase (LDH) level was also high at 535 U/I (normal, 124.0–232.0). The patient's basal levels of anterior pituitary hormones were almost within normal range. Diabetes insipidus was suspected based on the clinical findings. The markers of infections, such as HIV, HBV, and HCV, were all negative. The level of serum interleukin-2 receptor (sIL-2R) was examined because of detected multiple lesions, and it increased to 7526 U/mL (normal, 190–650).

**Figure 1:**
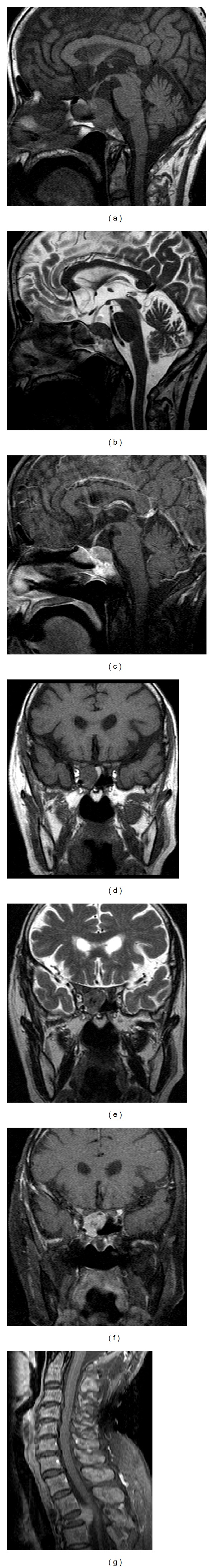
(a)–(f): Sagittal and coronal T1- ((a), (d)), T2- ((b), (e)), and Gd-enhanced T1-weighted ((c), (f)) MRI of the brain show a parasellar mass lesion. The lesion is isointense on T1-weighted images, iso- to hypointense on T2-weighted images, and inhomogeneously enhanced after contrast injection. (g) Sagittal Gd-enhanced thoracic MRI (*lower right*) shows a mass lesion with compression fracture of the Th3 vertebral body.

MR imaging of the brain revealed an intra- and parasellar mass lesion extending to the upper clivus, sphenoid sinus, and right cavernous sinus. The intrasellar mass compressed the pituitary gland to the dorsal side. The lesion was isointense on T1-weighted images, iso- to hypointense on T2-weighted images, and inhomogeneously enhancing after contrast injection (Figures 1(a)–1(f)). No other brain lesion was found. Thoracic MR imaging revealed a mass lesion with compression fracture of the Th3 vertebral body. It was isointense on T1- and T2-weighted images and homogeneously enhancing (Figure 1(g)). Fluorodeoxyglucose positron emission tomography (FDG-PET) imaging showed abnormal accumulations in the parasellar lesion and the C6 and Th3 vertebrae. Another workup had been done in a different hospital 2 weeks before the patient's admission at our facility, including computed tomography (CT) of the thorax, abdomen, and pelvis and bone scintigraphy; these were all negative. The preoperative differential diagnosis was metastatic tumor, malignant lymphoma, multiple myeloma, Tolosa-Hunt syndrome, Wegener granulomatosis, or skull base sarcoma.

We performed an endoscopic endonasal biopsy. The histological examination revealed hyperplastic tumor cells with a high mitotic index. Immunohistochemically, CD20 (a B-cell marker) was positive, CD3 (a T-cell marker) was negative, and the MIB-1 index was 100%. These findings were consistent with a diffuse large B-cell lymphoma. The bone marrow aspiration revealed positive lymphoma cells. A lumbar puncture showed no tumor cells in the cerebrospinal fluid. Another CT scan of the thorax, abdomen, and pelvis was performed, confirming the presence of hepatosplenomegaly, which seemed to be the consequence of infiltration of the tumor cells. Based on the results of the bone marrow aspiration and CT findings, this patient was diagnosed as having SPL.

**Figure 2:**
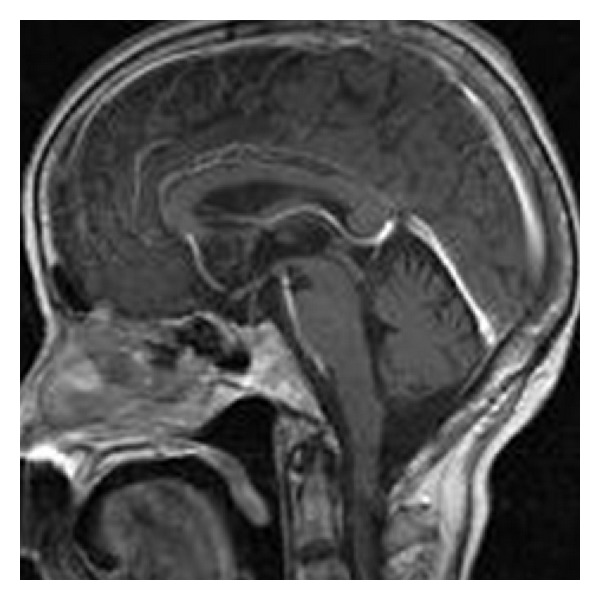
Sagittal Gd-enhanced brain MRI after 5-course chemotherapy shows complete disappearance of the tumor.

Postoperatively, the patient underwent five courses of chemotherapy with the R-THP-CVP regimen (rituximab, pirarubicin, doxorubicin, hydrochloride, cyclophosphamide hydrate, vindesine sulfate, and prednisolone). It took three weeks for one course of chemotherapy. After this chemotherapy, the follow-up MR imaging demonstrated a significant interval decrease in the size of the suprasellar and vertebral masses (Figure 2). Bone marrow aspiration revealed no infiltration. The serum levels of LDH and sIL-2R were decreased (LDH 156, sIL-2R 1063). She was discharged from our hospital after 4 months from operation. After discharge from our hospital, she received one more course of chemotherapy in another hospital. The patient no longer presented with fever. Her diabetes insipidus is well controlled with DDAVP. MR imaging showed complete remission and no signs of recurrence at 4 years after the operation.

## 3. Discussion

To our knowledge, only 19 cases of PPL and 16 cases of SPL have been reported. In these reports, several examinations including whole-body CT, gastrofiberscopy, bone marrow aspiration, and lumbar puncture were recommended to rule out another systemic lesion when a patient has PPL or SPL. This process is also important to differentiate between PPL and SPL. The clinical and radiological features of reported PPL and SPL are summarized in Tables 1 and 2. The mean age of the patients with PPL and SPL is almost the same. Male preponderance is apparent in the reported patients with PPL or SPL. Histologically, monoclonal B-cell non-Hodgkin lymphomas are the majority of PPLs and SPLs, similar to primary central nervous system (CNS) lymphomas [6, 10]. There is little difference in clinical presentation between PPL and SPL. Although the most common presentation is a pituitary insufficiency in both of these lymphomas, hypopituitarism is seen more often in PPL (73.7%) than SPL (41.2%), but there is no statistically significant difference. On the other hand, the frequency of diabetes insipidus is the same in both patient populations (PPL 36.8%, SPL 47.0%). Symptoms of fever, night sweat, and weight loss are more common in SPL (58.8%) than PPL (42.1%). These are the factors of unfavorable prognosis in CNS lymphoma [26]. As shown in Tables 1 and 2, patients with SPL tend to have longer overall survival than patients with PPL.

Unfortunately, regardless of PPL or SPL, the differential diagnosis is usually difficult, although prompt diagnosis and the initiation of treatment are crucial to achieve better clinical prognoses in patients with malignant lymphomas. The characteristic findings in MR imaging of pituitary lymphomas are isointense on T1-weighted images and iso- to hypointense on T2-weighted images [6, 8, 31] and homogeneous enhancement after Gd administration. Hyperintensity on diffusion weighted images is also typical, which reflects the hypercellular density of these tumors [6]. However, these radiological findings are not specific for pituitary lymphoma.

Aside from radiological findings, it is well known that the levels of serum soluble interleukin-2 receptor (sIL-2R) are elevated in patients with malignant lymphoma. However, there is only one reported case of systemic pituitary lymphoma in which the sIL-2R level was measured and elevated [30]. In our patient, the sIL-2R level was remarkably elevated, and it reminded us of a pituitary lymphoma. Of course, elevated sIL-2R is not a specific finding of malignant lymphoma; sIL-2R is also elevated in cases of autoimmune (e.g., rheumatoid arthritis) and other malignant diseases. Further, sIL-2R does not elevate in all cases of malignant lymphoma. Despite these limitations, elevated sIL-2R in a patient with a pituitary lesion can be a clue to the diagnosis of this rare disease. Although transsphenoidal biopsy is necessary in most cases to diagnose a pituitary lymphoma, these radiological and serological findings can contribute to a prompt diagnosis and induction of therapy.

For the treatment of primary CNS lymphomas, chemotherapy with the combined use ofa cyclophosphamide, doxorubicin, vincristine, and prednisone (CHOP) regimen, methotrexate (MTx), and/or rituximab has been commonly performed, but over the past decade, high-dose MTx therapy has become more common because of its penetrative tendency through the blood-brain barrier [12]. As shown in Tables 1 and 2, there are multiple reported cases treated with MTx (including intrathecal MTx), and the SPL patients did not have good outcome regardless of MTx treatment. Thus, it is also important to differentiate PPL from SPL when choosing a chemotherapy regimen. In the present SPL case, high-dose MTx was avoided, and a modified CHOP regimen and rituximab (R-THP-CVP) were used. With this chemotherapy, complete remission was achieved and no recurrence was detected at 47 months after the initial diagnosis.

In conclusion, we have described a case of SPL which was successfully treated with chemotherapy. Transsphenoidal biopsy was required because the differential diagnosis was difficult based on only radiological and clinical findings. However, T2 hypointensity in MR imaging and an elevated sIL-2R level can be useful to diagnose this disease, although these findings are not always specific. Neurosurgeons should be aware of this rare disease in the differential diagnosis of sellar lesion, because prompt diagnosis and treatment are crucial to achieve better clinical outcomes.

## Figures and Tables

**Table 1:** Clinical summary of 19 patients with PPL.

Age, sex	Clinical presentation	Endocrine abnormality	Pathology	Therapy	OS (months)
28, M [16]	HA, CN II/V	—	B-cell	Chemo + Rd	6, alive
49, M [12]	HA, CN VIII, Nys	Hypo, DI, PRL	B-cell	Ste	n.d.
48, M [4]	HA, CN VI, F, S&WL, nausea	—	B-cell	Antituberculous therapy	0.3, dead
73, F [13]	HA, fatige, CN VI	Hypo, DI, PRL	B-cell	Ste + Rd	21.6, alive
53, M [11]	HA, CN VI	—	T-cell	Rd	18, alive
48, M [3]	HA, CN III/VI	—	B-cell	n.d.	n.d.
67, F [9]	CN II/III	Hypo	B-cell	Rd	4M, alive
82, M [1]	HA, CN II	Hypo, DI	B-cell	Rd	n.d.
65, M [10]	n.d.	Hypo, PRL	B-cell	Chemo	0.5, alive
44, M [15]	HA, CN II	—	B-cell	Chemo + MTx + Rd	n.d.
86, F [31]	F, S&WL	Hypo, DI	B-cell	Chemo	3, dead
15, M [14]	F, S&WL	Hypo, DI	B-cell	Chemo + HD MTx	17, alive
74, M [8]	CN II, F, S&WL, mental status change	Hypo,	B-cell	Rd	1.5, dead
65, M [8]	CN VI, retrobulbar pain	Hypo,	B-cell	Chemo	24, dead
64, F [7]	nausea, diarrhea, edema	Hypo, DI	B-cell	Chemo + Rd	15, dead
47, M [5]	F, S&WL	Hypo,	T-cell	Chemo + i.t. MTx + Rd	5, alive
26, M [6]	HA, CN VI, F, S&WL	Hypo, PRL	NK/T-cell	Chemo + i.t. MTx + Rd	6, dead
49, F [2]	HA, F, S&WL	Hypo, DI, PRL	B-cell	MTx	48, alive
26, F [17]	HA, F, S&WL	Hypo, PRL	B-cell	—	0.5, dead

Hypo: anterior hypopituitarism; B-cell: B-cell lymphoma; Chemo: chemotherapy; CN: cranial nerve palsy; DI: diabetes insipidus; F, S&WL: fever, night sweat, and weight loss; HA: headache; HD: high dose; i.t.: intrathecal; MTx: methotrexate; n.d.: not described; NK: NK cell lymphoma; Nys: nystagmus; OS: overall survival; PRL: hyperprolactinemia; Rd: radiotherapy; Ste: steroid; T-cell: T-cell lymphoma; —: none.

**Table 2:** Clinical summary of 17 patients with SPL.

Age, sex	Clinical presentation	Endocrine abnormality	Pathology	Therapy	OS (month)
47, M [21]	HA, CN VI	Hypo,	HL	Chemo + Ste + Rd	4.5, alive
19, M [25]	HA, F, S&WL, dyspnea, seizure	Hypo, DI	C	Chemo + Ste	18, dead
56, M [24]	CN III	Hypo,	NHL	Chemo + MTx + Rd	25, alive
56, F [28]	F, S&WL, anorexia	DI, PRL	T-cell	Chemo + Ste	4.5, dead
50, M [22]	F, S&WL	—	T-cell	Chemo	72, alive
33, M [18]	F, S&WL, CN III/IV/VI	—	HL	Chemo	62, alive
70, F [29]	F, S&WL	Hypo,	—	—	1.75, dead
77, M [26]	Weakness	Hypo,	NHL	Ste	2.25, dead
64, M [27]	Abdominal pain	DI	B-cell	Chemo + MTx	36, alive
37, M [19]	HA	DI	B-cell	Chemo + i.t. MTx	10, alive
72, F [10]	F, S&WL, weakness	Hypo,	B-cell	Chemo	12, alive
70, M [30]	F, S&WL	SIADH	B-cell	Chemo + Ste	n.d., dead
59, M [32]	HA, CN III	—	B-cell	Chemo + i.t. MTx	18, alive
53, M [32]	HA, F, S&WL	DI	B-cell	Chemo + i.t. MTx + Rd	5, alive
41, M [23]	HA, F, S&WL, CN II	DI	T-cell	Chemo + Rd	18, dead
70, F [33]	HA, CN III	Hypo, DI	B-cell	—	n.d.
78, F (Our case)	F, S&WL	DI	B-cell	Chemo + Rd	48, alive

B-cell: B-cell lymphoma; Chemo: chemotherapy; CN: cranial nerve palsy; DI: diabetes insipidus; F, S&WL: fever, night sweat, and weight loss; HA: headache; HL: Hodgkin lymphoma; i.t.: intrathecal; n.d.: not described; NHL: non-Hodgkin lymphoma; OS: overall survival; Rd: radiotherapy; SIADH: syndrome of inappropriate secretion of ADH; Ste: steroid; T-cell: T-cell lymphoma; —: none.
